# Interleukin-6 actions in the hypothalamus protects against obesity and is involved in the regulation of neurogenesis

**DOI:** 10.1186/s12974-021-02242-8

**Published:** 2021-08-31

**Authors:** Vanessa C. Bobbo, Daiane F. Engel, Carlos Poblete Jara, Natalia F. Mendes, Roberta Haddad-Tovolli, Thais P. Prado, Davi Sidarta-Oliveira, Joseane Morari, Licio A. Velloso, Eliana P. Araujo

**Affiliations:** 1 Nursing School, University of Campinas, Campinas, Brazil; 2 Laboratory of Cell Signaling, University of Campinas, Rua Cinco de Junho, 350, Cidade Universitária, Campinas, SP 13083-877 Brazil

**Keywords:** Cytokine, Metabolism, Neuron, Astrocyte, Brain, Diabetes

## Abstract

**Background:**

Interleukin-6 (IL6) produced in the context of exercise acts in the hypothalamus reducing obesity-associated inflammation and restoring the control of food intake and energy expenditure. In the hippocampus, some of the beneficial actions of IL6 are attributed to its neurogenesis-inducing properties. However, in the hypothalamus, the putative neurogenic actions of IL6 have never been explored, and its potential to balance energy intake can be an approach to prevent or attenuate obesity.

**Methods:**

Wild-type (WT) and IL6 knockout (KO) mice were employed to study the capacity of IL6 to induce neurogenesis. We used cell labeling with Bromodeoxyuridine (BrdU), immunofluorescence, and real-time PCR to determine the expression of markers of neurogenesis and neurotransmitters. We prepared hypothalamic neuroprogenitor cells from KO that were treated with IL6 in order to provide an ex vivo model to further characterizing the neurogenic actions of IL6 through differentiation assays. In addition, we analyzed single-cell RNA sequencing data and determined the expression of IL6 and IL6 receptor in specific cell types of the murine hypothalamus.

**Results:**

IL6 expression in the hypothalamus is low and restricted to microglia and tanycytes, whereas IL6 receptor is expressed in microglia, ependymocytes, endothelial cells, and astrocytes. Exogenous IL6 reduces diet-induced obesity. In outbred mice, obesity-resistance is accompanied by increased expression of IL6 in the hypothalamus. IL6 induces neurogenesis-related gene expression in the hypothalamus and in neuroprogenitor cells, both from WT as well as from KO mice.

**Conclusion:**

IL6 induces neurogenesis-related gene expression in the hypothalamus of WT mice. In KO mice, the neurogenic actions of IL6 are preserved; however, the appearance of new fully differentiated proopiomelanocortin (POMC) and neuropeptide Y (NPY) neurons is either delayed or disturbed.

**Supplementary Information:**

The online version contains supplementary material available at 10.1186/s12974-021-02242-8.

## Background

Interleukin-6 (IL6) is a rather unique cytokine that exerts pleiotropic actions in distinct organs and systems [1, 2]. Factors such as magnitude of production, duration of response and site of action may have either protective or damaging impact on organism health [3, 4]. For example, the rapid activation of IL6 response during early infection has critical role in host protection [5–7], whereas in chronic inflammatory diseases and obesity-associated metabolic inflammation, the enduring actions of IL6 promote structural and functional losses that may result in irreversible damage [8–12].

One remarkable advance in the understanding of the beneficial actions of IL6 was the characterization of its production by the exercised muscle [13]. Differently of the pattern of production in infectious and chronic inflammatory conditions, during exercise, IL6 is produced for a short period of time, independently of a previous stimulation by tumor necrosis factor-alpha (TNFα) and accompanied by only moderate/low increase of other inflammatory substances [14]. This particular mode of IL6 production has been shown to mediate some of the health promoting actions of exercise, such as increasing systemic insulin action [15–17], reducing hepatic steatosis [18], and reducing hepatic glucose production [17].

The brain is an important site of action of IL6. Studies have shown that exercise-induced IL6 can attenuate memory impairment in models of Alzheimer’s disease [19, 20], whereas in the hypothalamus, IL6 produced in response to exercise can reduce diet-induced inflammation and correct the abnormal regulation of food intake [21, 22]. The reduction of neuroinflammation is one of the mechanisms mediating the actions of IL6 in models of exercise [21]; however, recent studies have shown that induction of neurogenesis is yet another important mechanism mediating the actions of IL6 improving cognition in models of traumatic brain injury and Alzheimer’s disease [23, 24].

Most studies evaluating adult neurogenesis have focused on the subventricular and subgranular zones (SVZ and SGZ, respectively), which provide new neurons for these specific regions, as well as for neighboring areas, such as striatum in humans [25, 26].

However, evidence suggests that replacement of hypothalamic neurons during life relies on local production, thus placing the hypothalamus as an autonomous adult neurogenesis niche [27, 28]. In concern with the hypothalamic functions controlling food intake and energy homeostasis, stimuli such as leptin and insulin, as well as nutrients, have been shown to regulate hypothalamic neurogenesis [28–31]. As IL6 produced during exercise is known to mitigate diet-induced hypothalamic dysfunction [21] and, considering that IL6 neurogenic potential has been shown in other neurogenic niches of the brain, here, we decided to evaluate the putative neurogenic actions of IL6 in the hypothalamus.

## Methods

### Experimental animals

Six- to 12-week-old male Swiss mice and 8-week-old male and female C57BL/6J mice (WT; Jackson Laboratory stock #000664) were obtained from the University of Campinas Animal Facility. Male and female, 8-week-old interleukin-6 knockout (KO; C57BL6/J KO^il6−/−^; Jackson Laboratory stock # 002254) were obtained from the Ribeirão Preto Medical School. Male and female, C57BL/6J and C57BL6/J KO^il6−/−^ pups (postnatal day 0–7) were obtained from the University of Campinas Animal Facility. All mice were kept in individual cages at 21 ± 0.5 °C, in 12/12-h light/dark cycle, with water and chow available ad libitum. In all experiments, control and intervention group mice were submitted to the same experimental settings.

### Experimental protocol

#### In vivo protocol #1

**Fig. 1:**
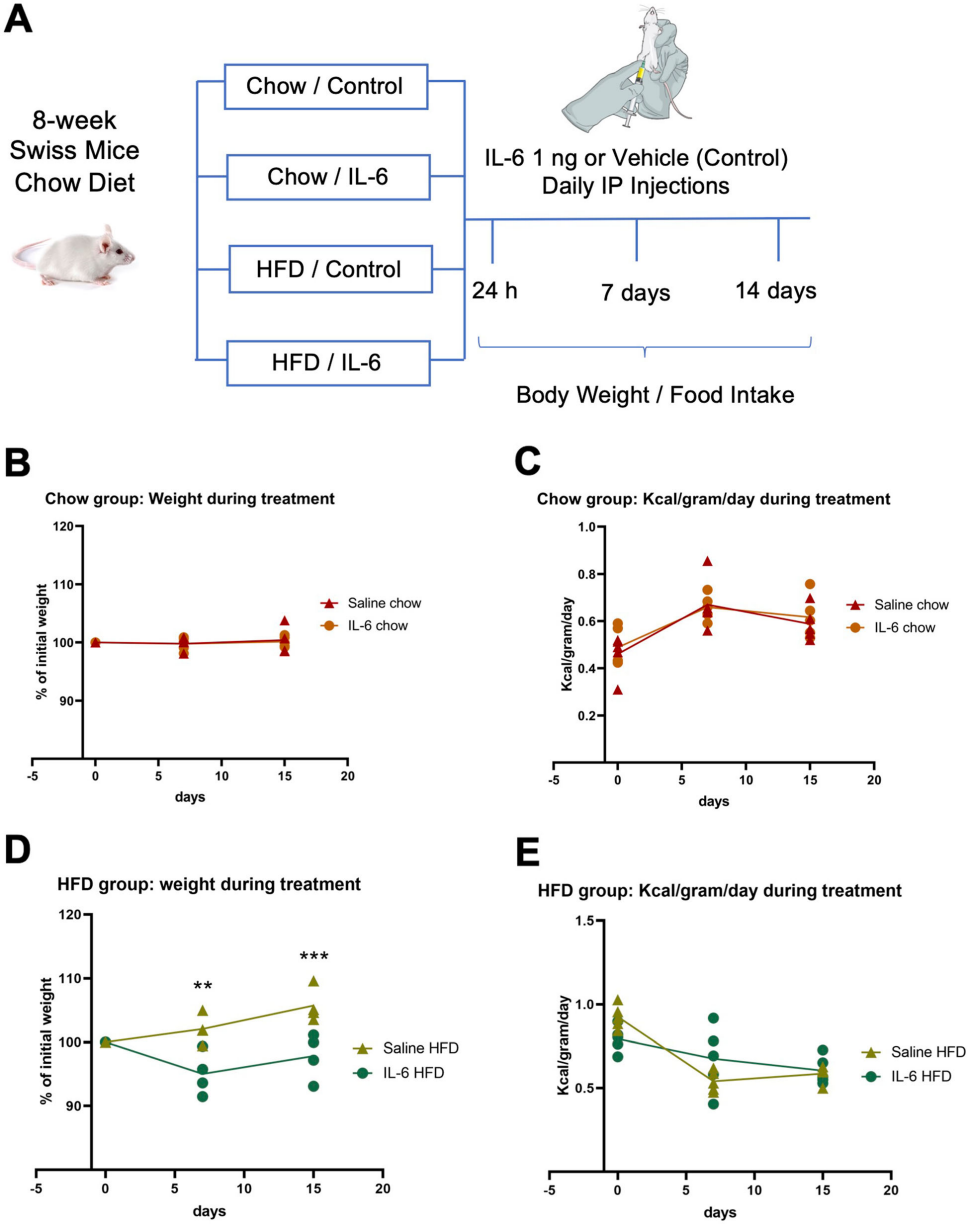
Effect of exogenous IL6 on caloric intake and body mass. Wild-type mice were fed on chow or high-fat diet (HFD) and treated intraperitoneally (IP) with exogenous IL6 or saline as depicted in A. Body mass (**B**, **D**) and caloric intake (**C**, **E**) were determined in mice fed chow (**B**, **C**) and HFD (**D**, **E**). In all experiments, *n* = 5 mice/group. Data was analyzed using two-way ANOVA with repeated measures and Bonferroni post-test. **p < 0.01; ***p < 0.001

Swiss mice were randomly separated into two groups: IL6 treatment or vehicle (saline). Thereafter, mice were fed a HFD for 2 weeks as described at the end of this paragraph (schematically illustrated in Fig. 1A). To determine caloric intake, the number of daily calories ingested by each mouse was divided by its weight in grams (Kcal/g/day); this approach was adopted because of the considerable variability in body mass among mice. The macronutrient composition of standard chow was 20 g% protein, 76 g% carbohydrate, and 4 g% fat, resulting in energy value of 17.5 kJ/g; the macronutrient composition of HFD was 20 g% protein, 45 g% carbohydrate, and 35 g% fat, resulting in energy value of 24.1 kJ/g.

#### In vivo protocol #2

Another set of Swiss mice were fed a HFD for 24 h and then defined as H (25% of animals that consumed highest amount of diet) or L (25% of animals that consumed lowest amount); thereafter, mice were euthanized and the hypothalami were collected for gene expression analysis (schematically illustrated in Fig. 3A).

#### In vivo protocol #3

In order to perform central delivery of palmitate (30 μM, 2 μl; Sigma) or vehicle (saline), Swiss mice were submitted to a single stereotaxic injection in the third ventricle under xylazine (10mg/kg, ip) and ketamine (100 mg/kg, ip) anesthesia. The coordinates from Bregma were as follows: anteroposterior, 0.0 mm; lateral, 0.0 mm; and depth, 4.5 mm [32, 33]. According to a time-course, mice were euthanized and the hypothalami were collected for gene expression analysis.

#### In vivo protocol #4

C57BL/6J mice were treated with vehicle (saline) or recombinant IL6 (Sigma) (1 ng ip, once a day, for up to 7 days, proliferation assay, or 14 days, survival assay). Bromodeoxyuridine (BrdU; Sigma) was used to evaluate cell proliferation. BrdU is a thymidine analog that is incorporated into the DNA double-helix during the S-phase of the cell cycle, and thus labels actively proliferating cells [34]. Mice received BrdU (0.1 M PBS, pH = 7.2; 50 mg/Kg 2x day ip for 7 days concomitant with IL6) and were euthanized 24 h after the last injection (proliferation assay, schematically illustrated in Fig. 4A) or 14 days after the last injection (survival assay, schematically illustrated in Fig. 4D).

#### In vivo protocol #5

Swiss mice were treated with a single injection of vehicle (saline) or recombinant IL6 (Sigma) (1 ng ip) at different time points (8 h, 6 h, 4 h, 2 h prior to extraction). Thereafter, mice were euthanized and the hypothalami collected for gene expression analysis (schematically illustrated in Fig. 5A).

#### In vivo protocol #6

Adult IL6 knockout (KO) mice fed on chow were compared to the respective WT, C57BL/6J controls. For that, we determined body mass and blood IL-6 levels at baseline conditions and 4 h after treatment with recombinant IL6 (Sigma) (1 ng ip) and hypothalamic gene expression analysis in baseline conditions.

### Immunofluorescence staining

Mice were deeply anesthetized with a solution of xylazine (10 mg/kg, ip) and ketamine (100 mg/kg, ip) and perfused through the left cardiac ventricle with 0.9% saline solution, followed by 4% paraformaldehyde (PFA) in 0.1M PBS (pH 7.4). After perfusion, the brains were removed, post-fixed in the same fixative solution for 24 h at room temperature (RT), and immersed in a 30% sucrose solution in PBS at 4 °C. Serial coronal sections (20 μm) of hypothalami were obtained with a cryostat (LEICA Microsystems, CM1860). In order to determine cell proliferation and survival in the hypothalamic ventricular zone and parenchyma, a series of one-in-four free-floating sections were processed for detection of the BrdU immunoreactivity. Differentiation was assessed by BrdU/neuronal nuclei (NeuN) double labeling. Briefly, after DNA denaturation in 2 N HCl at room temperature (RT) for 1 h and pre-incubation with 10% blocking solution (0.1M PBS with 10% normal goat or donkey serum and 0.2% Triton X-100), sections were incubated overnight at 4 °C in rat anti-BrdU (1:200; Ab6326) and mouse anti-NeuN (1:200; MAB377C3; Cy3 Conjugate) primary antibodies. The sections were then incubated with secondary antibody goat anti-rat FITC (1:200; sc2011) for 2 h at RT. All sections were mounted, cover slipped with Fluor Mount (Sigma), and stored at 4 °C. The morphological analyses were performed on coded slides, with the executing researcher blinded to the experimental group. The total numbers of BrdU-immunopositive cells in the HVZ and PA were estimated by manually counting all positive cells. From all sections containing the hypothalamus (1.06 to 2.30 mm posterior to Bregma), one-in-four series of sections were used for the analysis. The results were expressed as the counted number of labeled cells multiplied by 4 (the section interval), and the resulting number was corrected using the Abercrombie formula. Newly formed neurons (BrdU/NeuN-positive cells) were analyzed in one-in-four series of sections by immunofluorescence double staining [35]. The results were expressed as the counted number of labeled cells multiplied by 4 (the section interval), and the resulting number was corrected using the Abercrombie formula. The amount of BrdU/NeuN-positive cells was expressed as percentages. A maximum of 50 BrdU-labeled cells per mouse were randomly selected for analysis of co-labeling with NeuN. Double labeling was confirmed by three-dimensional orthogonal reconstruction (Imaje J software) of z-series (average of 18 images per series) of confocal microscopy covering the entire nucleus (or cell) of interest (confocal microscope Upright LSM780-NLO) in sequential scanning mode to avoid cross-bleeding between channels. Nuclei diameter was estimated using the Fiji software, as previously described [36]. Only cells that presented a typical aspect of NeuN surrounding the nucleus were considered. The representative images are the stack of the z-project of all images obtained within the 20 μm section. In order to avoid overestimation of cell count, we performed Abercrombie Correction according to the formula *N*= *n* × (*T*/(*T* + D), where *n* = cell counting, *T* = section thickness (20 μm), and *D* = estimated diameter of cell nuclei (approx. 15 μm), as described elsewhere [37, 38].

### Serum IL-6 determination

Blood samples were collected immediately after decapitation using a tube with citrate. Plasma was separated by centrifugation (1100×*g*) for 15 min at 4 °C and stored at − 80 °C until assay. IL-6 concentrations were determined using a commercially available Enzyme Linked Immunosorbent Assay kit, according to manufacturer instructions (Mouse IL-6 DuoSet ELISA, R&D Systems).

### Postnatal neurosphere culture

Postnatal day 0–7 pups were euthanized, their brains immediately removed, and the hypothalamus microdissected. Tissue fragments were repeatedly dissociated with a Pasteur pipette in PBS with 5.5 mM glucose, 100 U/mL penicillin, and 100 mg/mL streptomycin. Cells were suspended in 5 mL of proliferation media: Dulbecco’s modified Eagle’s medium (DMEM)-F12/Glutamax (Gibco) supplemented with growth factors (10 ng/mL human basic fibroblast growth factor, bFGF, and 10 ng/mL epidermal growth factor, EGF, sigma) 100 U/mL penicillin, 100 mg/mL streptomycin, and 1% B27 supplement. The floating neurospheres were allowed to grow in uncoated 25-cm^2^ flasks in a humidified incubator with a 5% CO_2_ atmosphere. On culture day 7, neurospheres were collected by centrifugation, mechanically dissociated using a pipette, and plated using approximately 100,000 cells per well in fresh proliferation medium, onto Poly-L-Lysine (PLL; Sigma)-coated 12 well culture plates for RNA extraction or glass coverslips for immunocytochemistry. Once the monolayer reached confluence, cell differentiation was induced by switching proliferation media for differentiation media: neurobasal medium (Gibco) supplemented with 1% B27 (Invitrogen), 500 mM/L-Glutamine (Sigma), and 50 units/mL penicillin/streptomycin (Life Technologies), without growth factors. The differentiation protocol was carried for 5 to 14 days to assess the differences between WT and KO mice and upon IL-6 (2 pg/ml, 20 pg/ml, 200 pg/mL, and 2 ng/mL) treatment over the expression of cell stemness and differentiation markers.

### Immunocytochemistry

Cover slips containing differentiated neurospheres were fixed with 4% PFA in 0.1 M PBS for 10 min at RT. After washing with PBS, the cells were incubated in blocking solution (10% normal donkey serum in 0.1 M PBS containing 0.2% Triton X-100) for 1 h at RT. The cells were then incubated in fresh blocking solution (3% normal donkey serum) containing rabbit anti-DCX (1:200; Cell Signaling 4,604), rabbit anti-MAP2 (1:200; ab32454), or anti-GFAP Cy3 (1:2000; ab49874) overnight at 4 °C. The cells were washed three times with PBS and incubated in blocking solution containing donkey anti-rabbit FITC (1:500; ab6798) for 1 h at RT, followed by DAPI for 10 min at RT. Following another three PBS washes, the slides were mounted using fluorescence mounting medium before image capturing on fluorescence microscopy (Olympus BX41). The results of immunopositive cells represent the average of 4 cover slips per experimental replicate, where 4–5 fields were imaged per cover slip and averaged. The number of immunopositive cells was quantified per image using the ImageJ software and are expressed as percentage relative to total nuclei.

### RNA extraction and quantitative real-time PCR

RNA samples were prepared using TRIzol (Invitrogen) according to the manufacturer’s recommendations. Spectrophotometry was employed for RNA quantification. For each sample, 2 μg of RNA was employed for the synthesis of complementary DNA (cDNA) using the High Capacity cDNA Reverse Transcription Synthesis kit (Applied Biosystems). Real-time PCR reactions were run using the TaqMan system (Applied Biosystems). Analyses were run using 4 μL (10 ng/μL) cDNA, 0.625 μL primer/probe solution, 1.625 μL H_2_O, and 6.25 μL 2X TaqMan Universal MasterMix. GAPDH (Mm99999915_g1) was employed as a reference gene. Gene expression was obtained using the StepOne Plus System software (Applied Biosystems). As negative control, no retrotranscriptase was added. All primers used in the study (Supplementary Table1) were certified for efficiency and specificity, as declared by the manufacturers. Nevertheless, we further validated the primers by amplifying the cDNA of each sample in triplicates at six different concentrations (3-fold serial dilutions). Both the primers for the target genes and reference gene were tested. The efficiency of the system was calculated using the formula: *E* = 10 (− 1/slope) − 1. The quantification method used for the calculations of the data was the 2^−dct^ method. Then, the average data from the control group was considered as 1, and other groups were relative to control. This was the fold change calculation used.

### Single-cell RNA sequencing data extraction and processing

scRNAseq data from the adult mouse arcuate nucleus was obtained from GEO (GSE93374). Data analysis was performed with Seurat v3.2.2 [39], a scRNAseq data analysis toolkit in R. Data was normalized with SCTransform, a negative binomial normalization approach to scRNAseq data [40]. We then selected top 5000 differentially expressed genes (DEGs) with a dispersion method and used these in the computation of dbMAP. dbMAP estimates data structure from a series of random walks by employing adaptive multiscale diffusion maps for a first round of dimensional reduction which is followed by an adapted UMAP implementation on the multiscale diffusion components and is available in python (10.2139/ssrn.3582067). Plots of dbMAP embeddings colored by annotated cell type were obtained with the function *DimPlot* of Seurat, with cell groupings from the original data report.

For Bayesian normalization of raw counts data, BASiCS (Bayesian Analysis of Single-Cell Sequencing data) was employed. Briefly, BASiCS is an integrated Bayesian hierarchical model that simultaneously performs data normalization and technical noise quantification to propagate statistical uncertainty. We used BASiCS new implementation which does not require the use of *Spike-ins* for data normalization and evaluates probabilistic consistency across batches instead [41]. Briefly, a Markov Chain Monte Carlo was constructed with the *BASiCS_MCMC* function of the BASiCS package in R with default recommended parameters and used for re-estimation of a denoised counts matrix with the *BASiCS_DenoisedCounts* function. All processed data and custom code used for analysis are available upon request.

### Visualization of gene expression

With the exception of plots from Suppl. Fig. 2, BASiCS probabilistic denoised gene expression was used for visualization. For dbMAP plots we used the *DimPlot* and *FeaturePlot* functions within Seurat, with the blend parameter set to *TRUE* for visualization of gene co-expression. All other visualizations were carried out with the Scanpy plotting API (10.1186/s13059-017-1382-0 ) after conversion to a *h5ad* AnnData object via SeuratDisk, an auxiliary Seurat package. Marker genes for main cell types defined on the original study were found via the Scanpy default workflow through Wilcoxon rank-sum tests and visualized in a heatmap.

### Statistical analysis

For all experiments, sample size was determined taking into consideration recommendations published elsewhere [42]. Data were analyzed using GraphPad Prism version 8.0.1. The statistical analyses were carried out using unpaired two-tailed Student’s *t*-test, one-way analysis of variance (ANOVA) with Tukey’s multiple comparison test, Kruskall-Wallis test with Dunn’s multiple comparison test, or two-way ANOVA with repeated measures and Bonferroni post-test when appropriate. Outliers were identified using Grubbs test. Data are presented as means ± standard error of the mean (SEM). A *p* value < 0.05 was considered to be statistically significant. Details of statistical analysis of all experiments described in this study are presented in Supplementary Table 2.

## Results

### Exogenous IL6 protects against diet-induced body mass gain

The consumption of large portions of dietary fats can induce hypothalamic inflammation, affecting the function and viability of critical neurons involved in the control of caloric intake and energy expenditure [43, 44]. Here, we asked if exogenous IL6 could act as a protective factor against diet-induced obesity. For that, 8-week mice were randomly divided into four groups as depicted in Fig. 1A (described in “In vivo protocol #1”). In mice fed chow, exogenous IL6 resulted neither in body mass (Fig. 1B) nor caloric ingestion (Fig. 1C) changes. However, in mice fed HFD, IL6 protected against body mass gain (Fig. 1D), which occurred independently of changes in caloric intake (Fig. 1E). The combined analysis of all groups confirmed the effect of IL6 protecting mice from diet-induced obesity (Suppl. Fig. 1A) without affecting caloric intake (Suppl. Fig. 1B).

### IL6 and its canonical receptor are expressed in the arcuate nucleus of the hypothalamus of adult mice

**Fig. 2:**
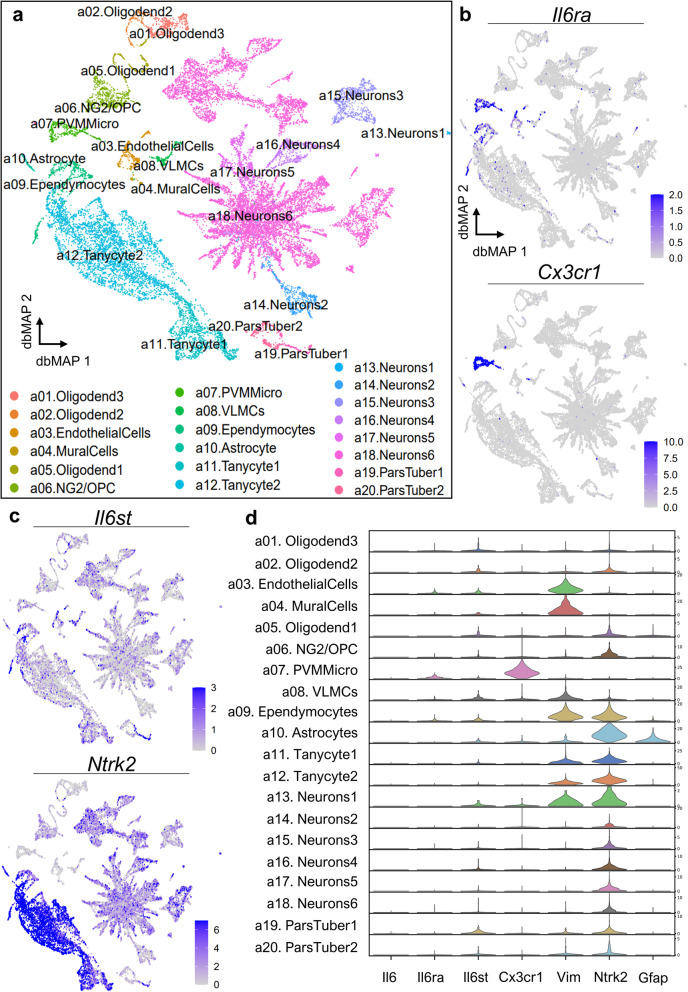
Analysis of single-cell RNA sequencing data reveal the expression pattern of IL6-receptor encoding genes in the arcuate nucleus of the hypothalamus. **A** Diffusion-based manifold approximation and projection (dbMAP) embedding of 20,921 single-cell transcriptomes of the arcuate nucleus of the hypothalamus, colored by their annotated cluster of origin. In this embedding, similar cells are mapped tightly, whereas dissimilar cells are mapped apart from each other, rendering clusters of cell types. Cells were annotated by their main cell type as defined in the original study. **B** (top) *Il6ra* mRNA expression, key-colored by expression intensity; *Il6ra* is found mainly in perivascular macrophages/microglia and ependymocytes and also in astrocytes and endothelial cells. **B** (bottom) *Cx3cr1* (a canonical microglial marker) mRNA expression, key-colored by expression intensity. **C** (top) *Il6st* mRNA expression, key-colored by expression intensity; *Il6st* is broadly expressed in the arcuate with some overlapping with the expression pattern of *Ntrk2* (bottom), which encodes a neurotrophic receptor. **D** Violin plot of the mRNA expressions of *Il6, Il6ra, Il6st, Cx3cr1, Vim, Ntrk2,* and *Gfap* in the arcuate hypothalamus, per main cell types. Oligodend, oligodendrocyte; NG2/OPC, oligodendrocyte progenitor cells; PVMMicro, perivascular macrophages/microglia; VLMCs, vascular and leptomeningeal cells; ParsTuber, *pars tuberalis*

Using dbMAP [45] as a dimensional reduction method for high-resolution visualization, we analyzed 20,921 public single-cell transcriptomes from the arcuate nucleus and median eminence of adult mice [46] into a comprehensive landscape of cellular heterogeneity. In this embedding (Fig. 2A), cells are colored by their original main cell type annotations. IL6 (*Il6*) mRNA was expressed in only a few cells, classified as microglia and tanycytes (Suppl. Fig. 2A). To exclude the possibility that *Il6-*expressing cells could be a result of random methodological noise, we performed a Bayesian normalization procedure in order to obtain a denoised, probabilistic-derived data matrix with BASiCS [47] and show that these cells indeed express barely detectable *Il6*, at much lower levels than corresponding marker genes (Suppl. Fig. 2A). Differently than *Il6*, its canonical receptor encoded by *Il6ra* and its trans-signaling receptor encoded by *Il6st* are abundantly expressed across different cell types (Fig. 2B, C and Suppl. Fig. 2B-2C). *Il6ra* is preferentially expressed in *Cx3cr1+* perivascular macrophages/microglia (cluster a07), endothelial cells (cluster a03), and ependymocytes (cluster a09), whereas *Il6st* is broadly expressed in tanycytes and arcuate neurons, as well as neurotrophic receptor *Ntrk2* and the neural stem cell marker *Nes* (Fig. 2B, C and Suppl. Fig. 2D-2F). Interestingly, *Nes* (a neural progenitor marker) and *Il6st* were co-expressed in tanycytes, which are currently held as the main hypothalamic neural stem cells [48]. *Il6st* mRNA was also detected in vascular leptomeningeal cells (a08. VLMCs), endothelial cells (a03), some neurons (a13, a16), and the *pars tuberalis* (a19) (Fig. 2D and Suppl. Fig. 2G). *Il6ra* mRNA expression pattern, on the other hand, was rather restricted to microglia (a07), ependymocytes (a09) and endothelial cells (a03) (Fig. 2D and Suppl. Fig. 2H) and so was *Il6ra* and *Il6st* co-expression (Suppl. Fig. 2I). The top five marker genes of each main cell population are shown on Suppl. Fig. 3.

### Hypothalamic IL6 is increased in mice genetically protected from diet-induced obesity

In outbred mice, body mass gain in response to HFD occurs according to a normal distribution [49]. We have previously shown that whenever outbred mice are fed on HFD, there is a direct correlation between caloric intake during the first 24 h following HFD introduction and body mass gain overtime [49]. Thus, mice presenting highest caloric intake are prone to obesity, whereas mice presenting lowest caloric intake are protected from obesity. Here, we asked if diet-induced obesity predisposition or protection is accompanied by differences in hypothalamic IL6 levels (described in “In vivo protocol #2”). Therefore, mice were submitted to the protocol depicted in Fig, 3A and the hypothalami were extracted 24 h after diet introduction. As shown in Fig, 3B, IL6 expression was increased in the hypothalamus of obesity-resistant mice.

### Palmitate induces a rapid increase in hypothalamic IL6 expression

**Fig. 3:**
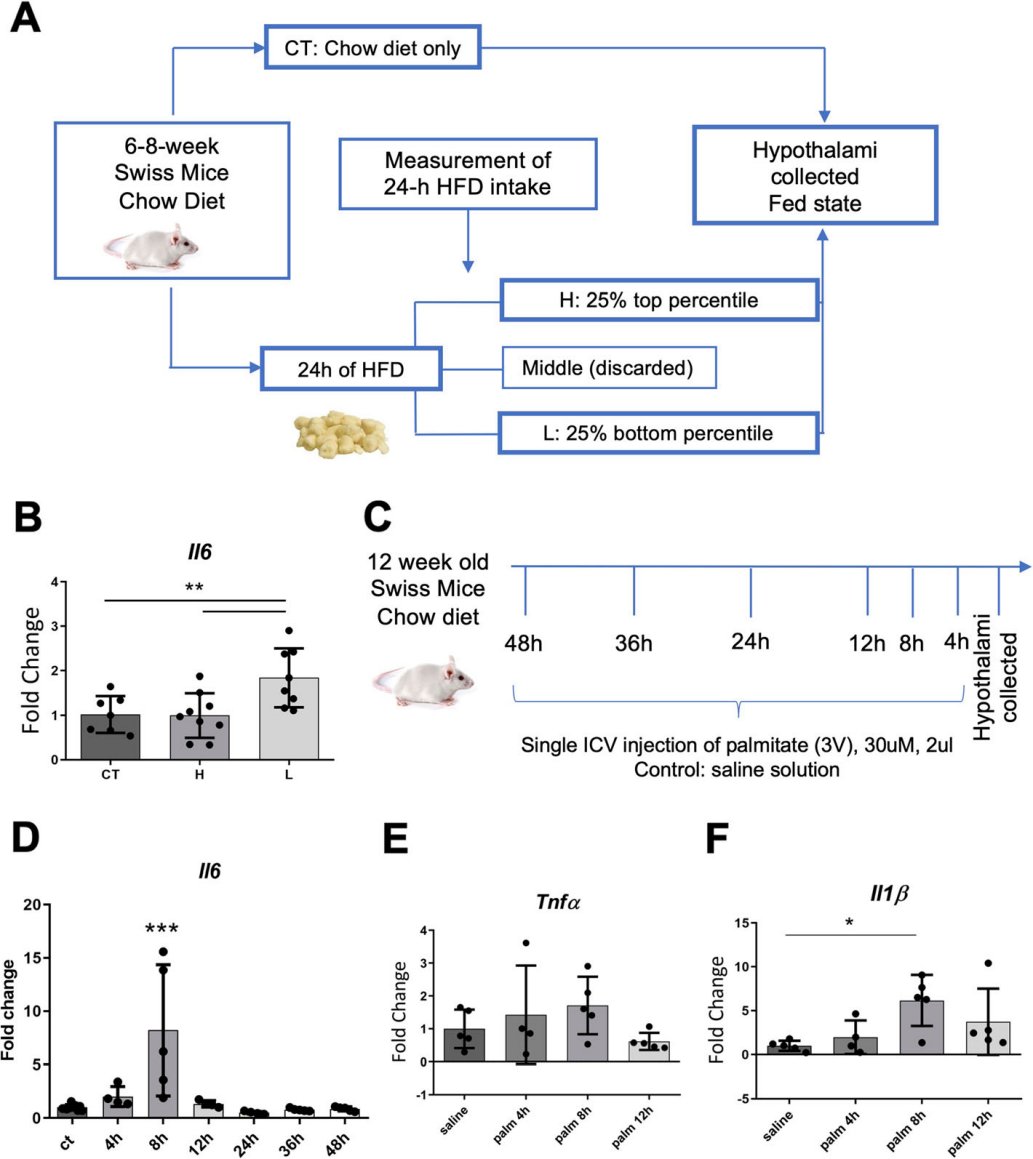
Hypothalamic transcript expression of IL6. In order to identify obese-prone (**H**) and obese-resistant (**L**) mice, we performed the procedure as depicted in A. The expression of hypothalamic IL6 was determined using real-time PCR (**B**). Palmitate was injected in a single intracerebroventricular (ICV) dose and hypothalamus was collected (**C**) for determinations of IL6 (**D**), TNFα (**E**), and IL1β (**F**) transcripts. **B**, *n* = 7–8 mice/group; **D**–**F**, *n* = 5 mice/group; *p < 0.05, **p < 0.01, ***p < 0.001. In bar graphs, results are presented as mean ± standard deviation

Palmitate is the predominant fatty acid in human diet and also in rodent HFD. It has been shown to cross the blood-brain barrier [50] and it is increased in the cerebrospinal fluid of obese humans [51]. Here, we asked if a direct injection of palmitate in the hypothalamus could increase the expression of IL6. For that, mice were acutely treated with a single intracerebroventricular (icv) dose of palmitate and the hypothalami were extracted for analysis (Fig. 3C) (described in “In vivo protocol #3”). As shown in Fig, 3D, there was a rapid increase in hypothalamic IL6 transcripts 8 h after treatment. Palmitate also promoted a trend to increase the transcripts expression of TNFα (Fig. 3E) and significantly increased the transcripts expression of IL1β in the hypothalamus (Fig. 3F).

### IL6 do not interfere in hypothalamic cell proliferation nor cell survival

**Fig. 4:**
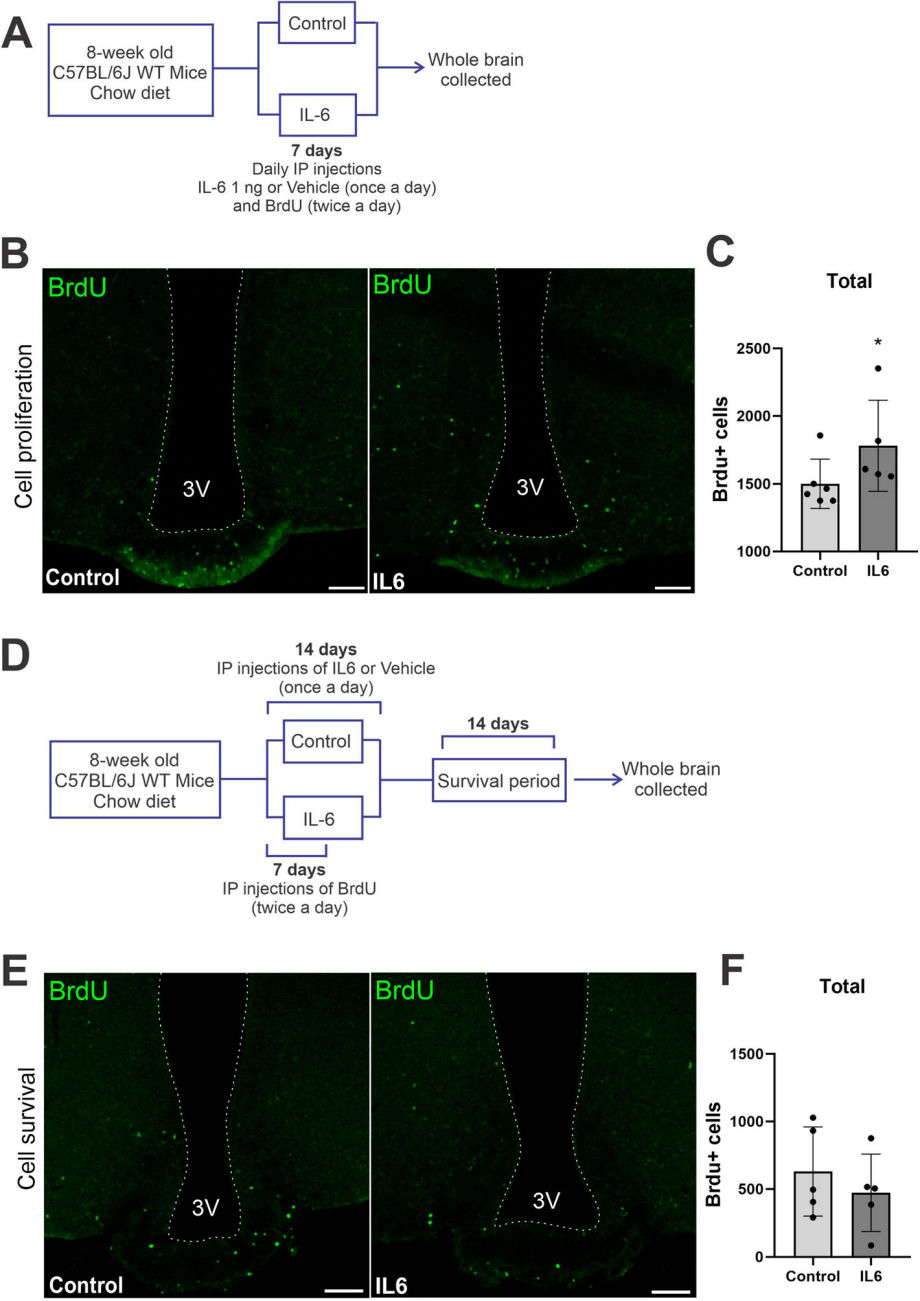
IL6 effect on hypothalamic cell proliferation and survival. Mice were treated with IL6 and BrdU according to the protocols as depicted in **A** and **D**. The mediobasal hypothalamus was prepared for analysis using immunofluorescence and confocal microscopy; cell counting was performed using 20× and 40× magnifications. Representative images are presented in **B** (proliferation assay) and **E** (survival assay). Total BrdU countings are presented in **C** (proliferation assay) and **F** (survival assay). In both assays, *n* = 6 mice/group. In bar graphs, results are presented as mean ± standard deviation

Next, we evaluated the capacity of exogenous IL6 to induce hypothalamic cell proliferation and survival. For that purpose, mice were submitted to two distinct protocols aimed at evaluating cell proliferation (Fig. 4A–C) and cell survival (Fig. 4D–F). At the end of the respective experimental periods, the brains were removed and used to determine the number of BrdU-positive cells during the 7- or 28-day time-windows (described in “In vivo protocol #4”). As depicted in the representative images of the hypothalamus (Fig. 4B, E) and in the graphical representation of cell counts (Fig. 4C, F), there were no significant differences between the groups.

### IL6 induces hypothalamic neurogenesis, as well as expression of neurogenesis-related transcripts in the hypothalamus

**Fig. 5:**
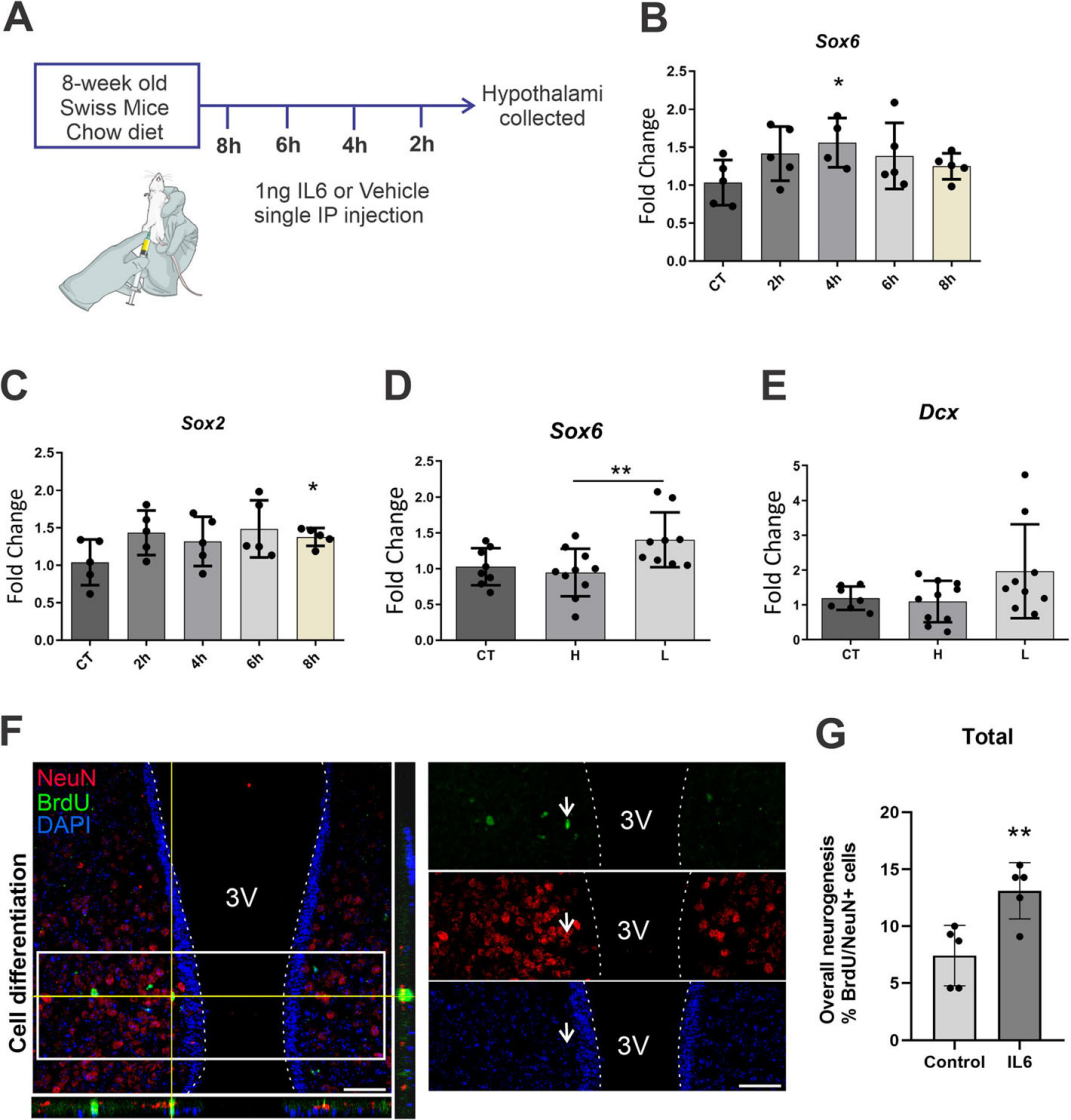
IL6 induces neurogenesis in the hypothalamus. Mice were treated with a single intraperitoneal dose of IL6, according to the protocol as depicted in **A**. The hypothalamic expressions of Sox6 (**B**) and Sox2 (**C**) were determined using real-time PCR. The transcript expressions of Sox6 (**D**) and doublecortin (**E**) were determined in the hypothalami collected from obese-prone (**H**) and obese-resistant (**L**) mice and treated with a single dose of IL6. **F** Mice were treated with IL6 and BrdU according to the protocols as depicted in Fig. 4D. The mediobasal hypothalamus was prepared for analysis using immunofluorescence and confocal microscopy; cell counting was performed using 20× and 40× magnifications. Total BrdU/NeuN counting is depicted in G (neurogenesis assay). **B**, **C** *n* = 5 mice/group; **D**, **E** *n* = 8–10 mice/group; **F**, **G**, *n* = 5 mice/group. *p < 0.05, **p < 0.01. In bar graphs, results are presented as mean ± standard deviation

Next, we evaluated the effect of IL6 to induce the expression of neurogenesis-related genes in the hypothalamus. For that, mice were treated with IL6 according to the protocol as depicted in Fig. 5A and the hypothalami were extracted for determination of gene expression (described in “In vivo protocol #5”). As shown in Fig. 5B and C, IL6 promoted increased expressions of Sox6 and Sox2 transcripts that encode transcription factors involved in neurogenesis, specifically in neural stem cells, during the proliferative state [52–54]. In addition, when looking into putative differences in the neurogenic response to IL6 comparing obesity-prone (H) and obesity-resistant (L) mice (described in “In vivo protocol #2”), we found that ip IL6 promoted a significantly larger expression of Sox6 transcripts (Fig. 5D) and a trend for increase of doublecortin transcripts (Fig. 5E) in obesity-resistant mice. Next, employing the same protocol as depicted in Fig. 4D (described in “In vivo protocol #4”, survival), we determined the number of BrdU-positive cells colocalizing with NeuN-positive cells. As shown in Fig. 5F and G, IL6 promoted an increased neuronal differentiation after 14 days, revealing its capacity to drive proliferative cells into a neuronal fate (*p* = 0.008, unpaired *T*-test, two-tailed). The total number of cells determined in this experiment are depicted in Suppl. Fig. 4.

### IL6 knockout mice are heavier and present a reduced expression of POMC in the hypothalamus

**Fig. 6:**
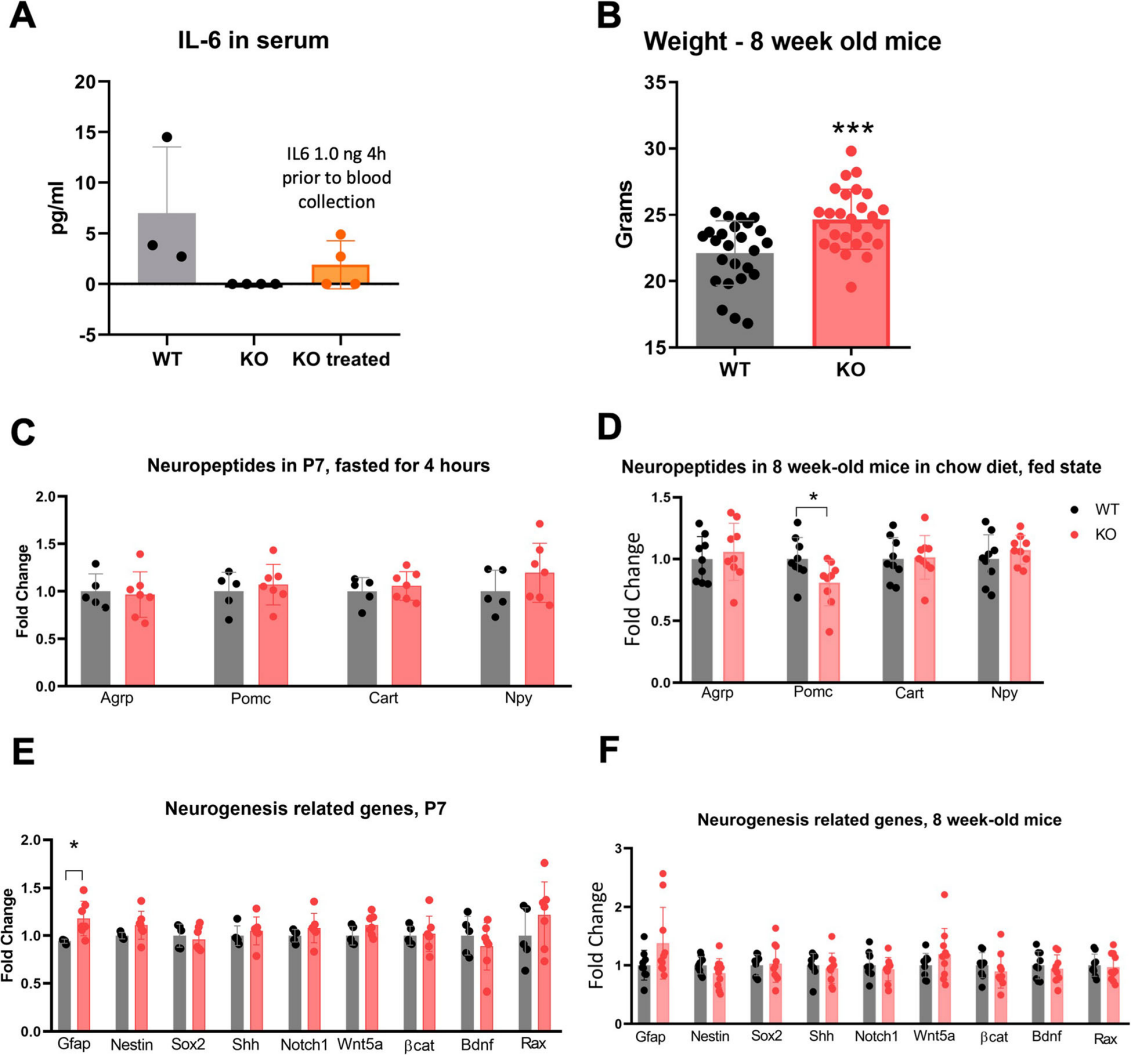
IL6 deficiency predisposes to obesity. IL6 was determined in the serum of wild-type (WT) and IL6 knockout (KO) mice treated or not with 1.0 ng exogenous IL6 (**A**). Body mass was determined at age 8 weeks (**B**). The expression of transcripts encoding for hypothalamic neurotransmitters (**C**, **D**) and neurogenesis-related genes (**E**, **F**) were determined using real-time PCR in hypothalamic samples collected in mice aged 7 days (**C**, **E**) or 8 weeks (**D**, **F**). **A** *n* = 3–4 mice/group; *n* = 25 mice/group; **C**, **E** *n* = 5–7 mice/group; **D**, **F** *n* = 9 mice/group. *p < 0.05, ***p < 0.001. In bar graphs, results are presented as mean ± standard deviation

Next, we evaluated body mass and hypothalamic gene expression in IL6 knockout (KO) mice (described in “In vivo protocol #6”). First, we tested if KOs were indeed completely depleted of circulating IL6, and if exogenous IL6 could promote a detectable increase of its blood levels. As shown in Fig. 6A, IL6 KO mice presented no detectable blood IL6 and the injection of 1 ng of IL6, 4 h prior to blood collection, resulted in the detection of blood IL6 in two out of four mice. At age 8 weeks, IL6 KO were heavier than controls (Fig. 6B), and at age 7 days, there were no differences in the expression of hypothalamic transcripts encoding for peptides related to energy homeostasis (Fig. 6C). However, at age 8 weeks, the expression of POMC was lower in IL6 KO as compared to control (Fig. 6D). Regarding transcripts related to neurogenesis, at age 7 days, IL6 KO presented lower levels of hypothalamic Gfap (Fig. 6E), whereas at age 8 weeks there were no differences between IL6 KO and control (Fig. 6F).

### IL6 increases markers of neurogenesis in hypothalamic neuroprogenitor cells

**Fig. 7:**
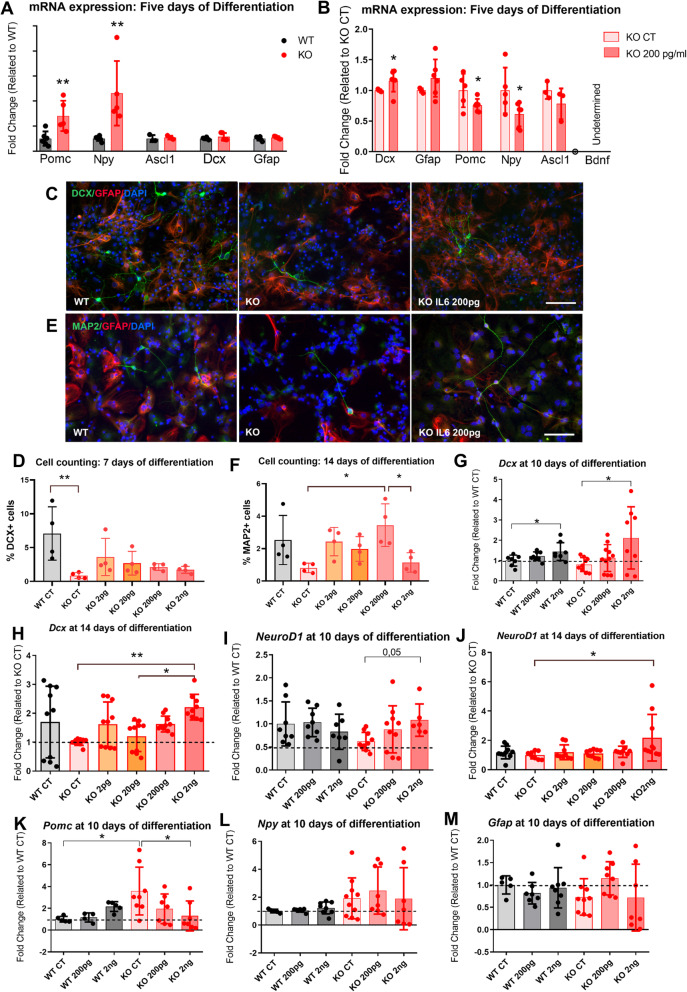
IL6 induce neurogenic factors expression in hypothalamic neuroprogenitor cells (NPCs). Transcripts were determined using real-time PCR in the NPCs of WT and IL6 KO mice after a 5-day differentiation period: first, WT and IL6 KO hypothalamic cells were analyzed in basal conditions (**A**), and then, IL6 KO NPCs were treated with exogenous IL6 in doses depicted (**B**). Representative immunocytochemistry images of 7-day differentiated cells (green, DCX; red, GFAP; blue, DAPI) (**C**) and DCX-positive cell counting (**D**), and representative immunocytochemistry images of 14-day differentiated cells (green, MAP2; red, GFAP; blue, DAPI) (**E**) and MAP2-positive cell counting (**F**). After 10- (**G**, **I**, **K**–**M**) or 14-day (**H** and **J**) differentiation period, transcripts were determined using real-time PCR in the NPCs of WT and IL6 KO mice treated with exogenous IL6 in doses depicted in the panels (G-M). **A**, **B** *n* = 12 (3–4 well/group, 2 independent experiments); **C**–**F** *n* = 24 (4 well/group, 2 independent experiments); **G**–**M** *n* = 48 (6–9 well/group, 3 independent experiments). *p < 0.05, **p < 0.01. In bar graphs, results are presented as mean ± standard deviation

Next, we employed neuroprogenitor cells (NPCs) obtained from IL6 KO mice. The cells were expanded using a method to generate neurospheres. Thereafter, NPCs were employed to determine the direct effect of IL6 to promote differentiation towards a neuronal fate. The expressions of Pomc and Npy transcripts were increased in the hypothalamic NPCs obtained from IL6 KO mice (Fig. 7A). When IL6 was added to culture media, there was an increased expression (mRNA and protein) of doublecortin and reduced mRNA expressions of Pomc and Npy (Fig. 7B–D). In addition, IL6 promoted a trend to increase the number of doublecortin positive cells (Fig. 7C, D) and a significant increase in the number of MAP2 positive cells (Fig. 7E, F). In NPCs differentiated for longer time (10–14 days), IL6 promoted increased expressions of doublecortin (Fig. 7G, H) and NeuroD1 transcripts (Fig. 7I, J), a reduction of Pomc (Fig. 7K), and no changes in Npy (Fig. 7L) and Gfap transcripts (Fig. 7M).

## Discussion

Adult neurogenesis warrants neuronal renewal throughout life [25]. It relies on the existence of neural stem/progenitor cells (NSPCs) that reside in a few brain niches and respond to distinct endogenous and exogenous stimuli, differentiating to integrate specific networks [55–59]. The SVZ and the SGZ are the most important neurogenic niches producing new neurons involved in a multitude of functions such as memory, cognition, and olfaction [60–62]. A smaller neurogenic niche exists in the hypothalamus and is responsible for the renewal of neurons involved in whole body energy homeostasis [27, 28].

Neurons of the mediobasal hypothalamus (MBH) control food intake and energy expenditure in response to signals delivered by hormones, neural inputs, and nutrients [44, 63–67]. In obesity and aging, hypothalamic inflammation leads to neuronal loss, which affects the balance between neuronal subpopulations exerting anabolic and catabolic functions [68–71]. Certain stimuli can promote a neurogenic response that results in the generation of neurons that could reestablish homeostasis [27, 28, 31]. Thus, it is believed that pharmacological and nutritional approaches leading to the generation of new hypothalamic neurons that counterbalance the effects of obesity and ageing could be useful in therapeutics [63, 72–74].

Here, we tested the hypothesis that IL6 could induce hypothalamic neurogenesis. The hypothesis was based in two premises: (i) IL6 produced in response to exercise improves memory and cognition, at least in part, due to its neurogenic roles in the hippocampus [19, 23] and (ii) IL6 produced in response to exercise reduce obesity-associated hypothalamic inflammation restoring the control of food intake and energy expenditure [21].

First, we showed that exogenous IL6 can reduce body mass gain in mice fed HFD but not in mice fed chow. These results are in concert with the fact that IL6 KO mice develop late-onset obesity and the replacement of exogenous IL6 corrects the phenotype [75]. Most of the effects of IL6 to reduce body mass depends on its action in the hypothalamus [75]. It is long known that both IL6 and its receptor are expressed in the hypothalamus [76]; however, with the recent advance in single-cell transcriptome technology, details regarding the expression of distinct transcripts can be traced to subpopulation levels [77]. To explore the expression profiles of *Il6* and *Il6ra* in the hypothalamus cellular landscape, we analyzed 20,921 public single-cell transcriptomes [46] with the dbMAP dimensionality reduction method [45]. This is the first single-cell analysis of IL6 and IL6 receptors in the hypothalamus, providing advance by showing that *Il6* is lowly expressed and restricted to microglia and tanycytes, whereas its canonical receptor *Il6ra* is consistently expressed in ependymocytes, astrocytes, endothelial cells, and particularly in the microglia. On the other hand, *Il6st*, which is needed for *Il6ra* signaling and might be directed activated by IL6 trans-signaling [78], is broadly expressed across almost all cell types, and particularly in neurons and tanycytes, although *Il6st* and *Il6ra* mRNA co-expression was detectable only in microglia and ependymocytes, suggesting classical IL6 signaling through IL6RA is restricted to these cell types in the arcuate hypothalamus.

In humans, exposure to environmental factors that predispose to obesity, results in variable body mass gain, which occurs according to a normal distribution [79]. This reflects the genetic nature of obesity-prone and obesity-resistant phenotypes [80]. Similar pattern occurs whenever outbred rodents are fed on HFD [49, 81]. Previously, we showed that rapid regulation of hypothalamic POMC following the introduction of HFD is an important factor determining predisposition to obesity [49, 70]. Here, we showed that obesity-resistant mice express larger amounts of IL6 than obesity-prone mice. These data provide additional support for the anti-obesity actions of IL6 [21, 75] and suggests that enhanced capacity to produce IL6 in the hypothalamus could be a genetically determined mechanism that protects against excessive body mass gain. This is further supported by human studies that show both obesity-protection [3] and obesity predisposition [82] associated with different polymorphisms of the IL6 gene.

Next, using a living-mouse model, we showed that ip IL6 was capable of increasing the number of newly generated neurons in the arcuate nucleus of the hypothalamus. This was accompanied by the increased expression of neurogenic-related transcripts Sox2 and Sox6. Moreover, we showed that the neurogenic response was more evident in obesity-resistant mice. This is the first evidence for a pro-neurogenic action of IL6 in the hypothalamus. There are few studies that have evaluated the potential implication of IL6 in adult neurogenesis and all of them were focused on the hippocampus. Some studies showed that chronically, IL6 could impair neurogenesis [83, 84]; however, two studies have shown that in the context of exercise, IL6 could promote neurogenesis impacting positively in memory and cognition [19, 23], whereas another study has shown an increase in hippocampal IL6 occurring in response to a neurogenic stimulus provided by marrow-derived mesenchymal stem cells [85].

Further insight into the roles of IL6 in hypothalamic neurogenesis was obtained determining the expression of neurogenic-related genes and neurotransmitters in IL6 KO mice. First, we reproduced results of previous studies showing that IL6 KO mice are obese-prone [75]. Next, we showed that in mice aged 7 days there was increased expression of hypothalamic Gfap, whereas in mice aged 8 weeks, there was a reduction of POMC. This could indicate an abnormal neurogenic process, since Gfap is a marker of both astrocytes and tanycytes, cells that are involved in hypothalamic neurogenesis [86, 87]. The reduction of POMC could result from the abnormal neurogenic process and contribute to the obese-prone phenotype of IL6-deficient mice [31, 88, 89].

In the last part of the study, we evaluated NPCs produced from IL6 KO mice hypothalami. Both proliferation rate and expression of POMC and NPY were increased at the baseline. Upon treatment with IL6, NPCs that differentiated for five days presented increased expression of doublecortin and decreased expression of POMC and NPY. Doublecortin is a microtubule-associated protein that expresses early during the development of neurons [90]; we reasoned that stimulation with IL6 could be inducing an upsurge of neurogenesis and only undifferentiated neurons could be detected at this time. Therefore, we increased differentiation time for 10 and 14 days; NPY expression was normalized, however, increased doublecortin persisted and was accompanied by similar changes in the expression of NeuroD1, another important factor associated with adult neurogenesis [91]. Nevertheless, the IL6-induced reduction of POMC persisted suggesting that in the IL6 deficient environment the stimulus with exogenous IL6 could generate an abnormal pattern of either survival or generation of specific neuronal populations.

Because of the functional nature of many neurons in the MBH, which are involved in the control of caloric intake and energy expenditure, it was expected that neurogenic stimuli affecting neurons of this region could impact on whole body energy homeostasis. In fact, we showed that exogenous IL6 reduces body mass, whereas IL6 deficiency results in obesity predisposition. Moreover, in IL6 KO mice, the hypothalamic transcript levels of POMC are reduced. At least in part, these effects could be attributed to the hypothalamic neurogenic actions of IL6. We acknowledge that studding the whole MBH and not specific cell subpopulations is a limitation of this study that could be further explored in the future.

## Conclusions

In this study, we provide the evidence for a role of IL6 in the regulation of hypothalamic neurogenesis. Both, in living mice and NPCs, IL6 stimulated cell proliferation and induced the expression of markers of immature neurons; however, in IL6 deficiency, exogenous IL6 administration seem to modify the pattern of differentiation of neurons. Further studies should define the mechanism linking IL6-induced neurogenesis with neuronal fate determination.

## Supplementary Information

**Additional file 1: Supplementary Figure 1.** Effect of exogenous IL6 on caloric intake and body mass. Wild-type mice were fed on chow or high-fat diet (HFD) and treated intraperitoneally (IP) with exogenous IL6 as depicted in Figure 1A. Body mass (A) and caloric intake (B) were determined in mice fed chow and HFD. In all experiments, n=5 mice/group. Two-way ANOVA with repeated measures and Bonferroni post-test was applied for the statistics of all data. *p<0.05, IL6 HFD vs. saline HFD (day 7 and day 14). *p<0.05, saline HFD vs. saline chow (day 14). *p<0.05, saline HFD vs. IL6 chow (day 14). **Supplementary Figure 2.** Bayesian analysis of single-cell RNA sequencing data and key genes coexpression patterns. A-F. dbMAP embeddings of the arcuate nucleus and median eminence scRNAseq data, colored by raw mRNA expression levels (left) or corresponding Bayesian probabilistic-denoised counts levels (right). Bayesian analysis was performed with BASiCS (Bayesian Analysis for Single-Cell Sequencing). G. dbMAP embedding key-colored by Nes (red) and Il6st (green) mRNA co-expression levels. H. dbMAP embedding key-colored by Cx3cr1 (red) and Il6ra (green) mRNA co-expression levels. I. dbMAP embedding key-colored by Il6ra (red) and Il6st (green) mRNA co-expression levels. **Supplementary Figure 3.** Heatmap of top five marker genes for the arcuate nucleus and median eminence main cell types. Heatmap showing mRNA expression of top five marker genes from the main cell types of the arcuate nucleus and median eminence. Marker genes were found with a Wilcoxon rank-sum test and then scaled. Z-scores are then used for coloring. Supplementary Figure 4. IL6 effect on hypothalamic cell proliferation and survival. Mice were treated with IL6 and BrdU according to the protocols as depicted in Fig. 4A and 4D. The mediobasal hypothalamus was prepared for analysis using immunofluorescence and confocal microscopy; cell counting was performed using 20X and 40X magnifications. Abercrombie corrected total BrdU countings are presented in A (proliferation assay) and B (survival assay). In both assays, n=6 mice/group). In bar-graphs results are presented as mean ± standard deviation. Supplementary Figure 5. Total number of cells determined in the experiment depicted in Figure 5G. Mice were treated with IL6 and BrdU according to the protocols as depicted in Figure 4D. The mediobasal hypothalamus was prepared for analysis using immunofluorescence and confocal microscopy; cell counting was performed using 20X and 40X magnifications. N=5 mice/group. **Supplementary Table 1.**. Detailed information of primers used in the study. All primers were purchased from Thermo Fischer Scientific. **Supplementary Table 2.** Raw data for statistical analysis.

## Data Availability

Data and materials are available upon request.

## Abbreviations

ASCL1: Achaete-scute homolog 1
BDNF: Brain-derived neurotrophic factor
BrdU: Bromodeoxyuridine
CEUA: Ethics committee on animal use
Ct: Control
Cx3Cr1: C-x3-c motif chemokine receptor 1
DCX: Doublecortin
DMEM: Dulbecco’s modified eagle medium
DNA: Deoxyribonucleic acid
EGF: Endotelial growth factor
FGF: Fibroblast growth factor
GAPDH: Glyceraldehyde 3-phosphate dehydrogenase
GFAP: Glial fibrillary acidic protein
HFD: High-fat diet
ICV: Intracerebroventricular
IL-1β: Interleukin-1β
IL-6: Interleukin-6
IL6ra: Interleukin-6 receptor subunit alpha
IL6st: Interleukin-6 cytokine family signal transducer
IP: Intraperitoneal
KO: Knockout
L: Low intake of high-fat diet
H: High intake of high-fat diet
HCl: Hydrochloric acid
MAP 2: Microtubule-associated protein 2
MBH: Mediobasal hypothalamus
mRNA: Messenger ribonucleic acid
NeuroD1: Neuronal differentiation
NeuN: Nuclear neuronal antigen
NPC: Neural progenitor cells
NSC: Neural stem cells
Ntrk2: Neurotrophic receptor tyrosine kinase 2
NPY: Neuropeptide Y
PBS: Phosphate saline buffer
PCR: Polymerase chain reaction
POMC: Proopiomelanocortin
RNA: Ribonucleic acid
SGZ: Subgranular zone
SOX: Sex determining region Y box
SVZ: Subventricular zone
TNF-α: Tumor necrosis factor α
WT: Wild-type
