# Supplementary material for: Interleukin-6 actions in the hypothalamus protects against obesity and is involved in the regulation of neurogenesis
Source: J Neuroinflammation. 2021 Aug 31;18:192. doi: 10.1186/s12974-021-02242-8 (PMC8408946; doi:10.1186/s12974-021-02242-8)
Supplement: Supplementary file 1 — Additional file 1: Supplementary Figure 1. Effect of exogenous IL6 on caloric intake and body mass. Wild-type mice were fed on chow or high-fat diet (HFD) and treated intraperitoneally (IP) with exogenous IL6 as depicted in Figure 1A. Body mass (A) and caloric intake (B) were determined in mice fed chow and HFD. In all experiments, n=5 mice/group. Two-way ANOVA with repeated measures and Bonferroni post-test was applied for the statistics of all data. *p<0.05, IL6 HFD vs. saline HFD (day 7 and day 14). *p<0.05, saline HFD vs. saline chow (day 14). *p<0.05, saline HFD vs. IL6 chow (day 14). Supplementary Figure 2. Bayesian analysis of single-cell RNA sequencing data and key genes coexpression patterns. A-F. dbMAP embeddings of the arcuate nucleus and median eminence scRNAseq data, colored by raw mRNA expression levels (left) or corresponding Bayesian probabilistic-denoised counts levels (right). Bayesian analysis was performed with BASiCS (Bayesian Analysis for Single-Cell Sequencing). G. dbMAP embedding key-colored by Nes (red) and Il6st (green) mRNA co-expression levels. H. dbMAP embedding key-colored by Cx3cr1 (red) and Il6ra (green) mRNA co-expression levels. I. dbMAP embedding key-colored by Il6ra (red) and Il6st (green) mRNA co-expression levels. Supplementary Figure 3. Heatmap of top five marker genes for the arcuate nucleus and median eminence main cell types. Heatmap showing mRNA expression of top five marker genes from the main cell types of the arcuate nucleus and median eminence. Marker genes were found with a Wilcoxon rank-sum test and then scaled. Z-scores are then used for coloring. Supplementary Figure 4. IL6 effect on hypothalamic cell proliferation and survival. Mice were treated with IL6 and BrdU according to the protocols as depicted in Fig. 4A and 4D. The mediobasal hypothalamus was prepared for analysis using immunofluorescence and confocal microscopy; cell counting was performed using 20X and 40X magnifications. Abercrombie corrected total BrdU [file 12974_2021_2242_MOESM1_ESM.docx]

Supplementary Data

Interleukin-6 actions in the hypothalamus protects against obesity and is involved in the regulation of neurogenesis

Vanessa C. Bobbo et al.

**Supplementary Figure 1. Effect of exogenous IL6 on caloric intake and body mass.** Wild-type mice were fed on chow or high-fat diet (HFD) and treated intraperitoneally (IP) with exogenous IL6 as depicted in Figure 1A. Body mass (A) and caloric intake (B) were determined in mice fed chow and HFD. In all experiments, n=5 mice/group. Two-way ANOVA with repeated measures and Bonferroni post-test was applied for the statistics of all data. *p<0.05, IL6 HFD vs. saline HFD (day 7 and day 14). *p<0.05, saline HFD vs. saline chow (day 14). *p<0.05, saline HFD vs. IL6 chow (day 14).

**Supplementary Figure 2. Bayesian analysis of single-cell RNA sequencing data and key genes coexpression patterns.** A-F. dbMAP embeddings of the arcuate nucleus and median eminence scRNAseq data, colored by raw mRNA expression levels (left) or corresponding Bayesian probabilistic-denoised counts levels (right). Bayesian analysis was performed with BASiCS (Bayesian Analysis for Single-Cell Sequencing). G. dbMAP embedding key-colored by *Nes* (red) and *Il6st* (green) mRNA co-expression levels. H. dbMAP embedding key-colored by *Cx3cr1* (red) and *Il6ra* (green) mRNA co-expression levels. I. dbMAP embedding key-colored by *Il6ra* (red) and *Il6st* (green) mRNA co-expression levels.

**Supplementary Figure 3. Heatmap of top five marker genes for the arcuate nucleus and median eminence main cell types.** Heatmap showing mRNA expression of top five marker genes from the main cell types of the arcuate nucleus and median eminence. Marker genes were found with a Wilcoxon rank-sum test and then scaled. Z-scores are then used for coloring.


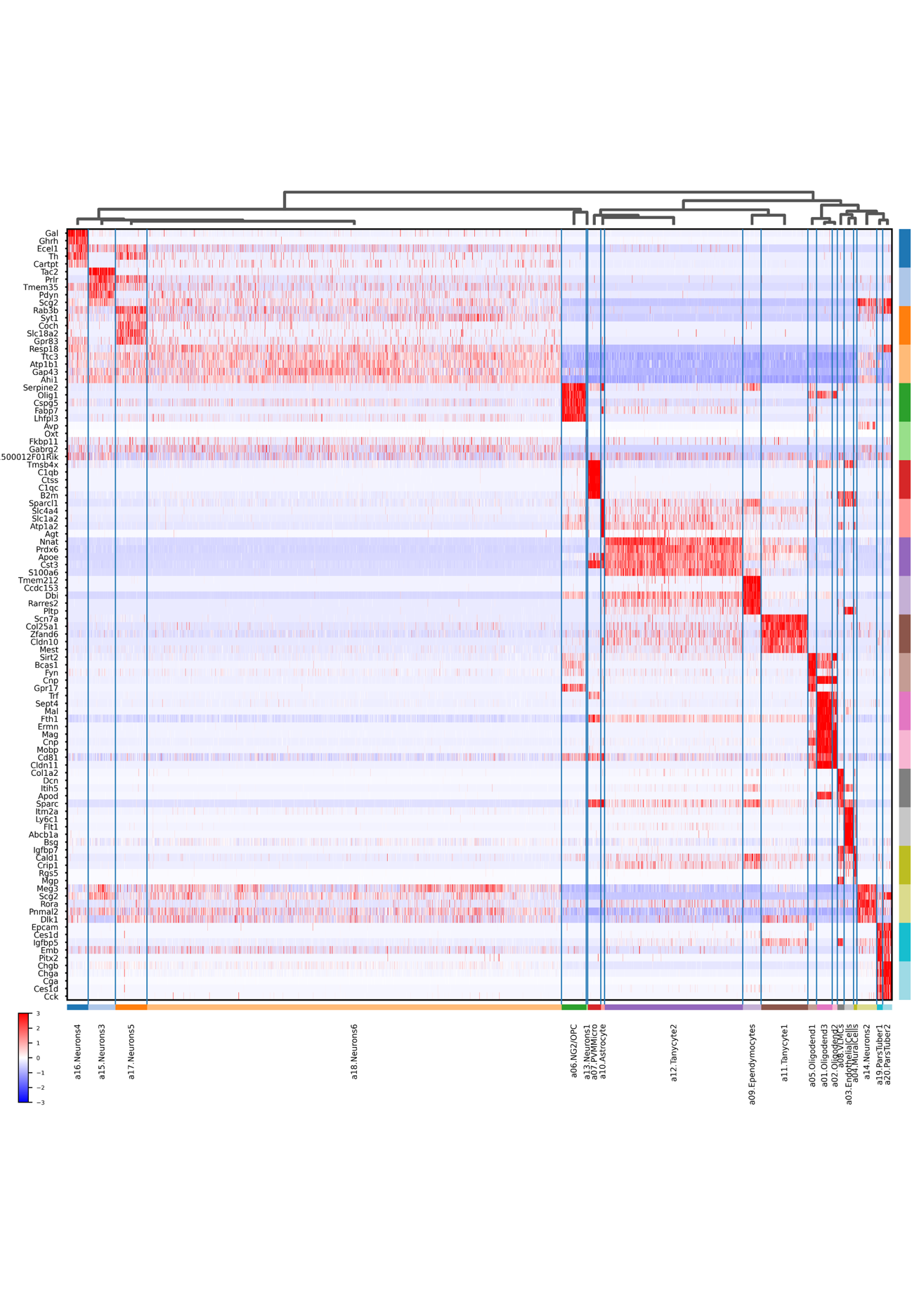


**Supplementary Figure 4. IL6 effect on hypothalamic cell proliferation and survival.** Mice were treated with IL6 and BrdU according to the protocols as depicted in Fig. 4A and 4D. The mediobasal hypothalamus was prepared for analysis using immunofluorescence and confocal microscopy; cell counting was performed using 20X and 40X magnifications. Abercrombie corrected total BrdU countings are presented in A (proliferation assay) and B (survival assay). In both assays, n=6 mice/group). In bar-graphs results are presented as mean ± standard deviation.

**Supplementary Figure 5. Total number of cells determined in the experiment depicted in Figure 5G.** Mice were treated with IL6 and BrdU according to the protocols as depicted in Figure 4D. The mediobasal hypothalamus was prepared for analysis using immunofluorescence and confocal microscopy; cell counting was performed using 20X and 40X magnifications. N=5 mice/group.

**
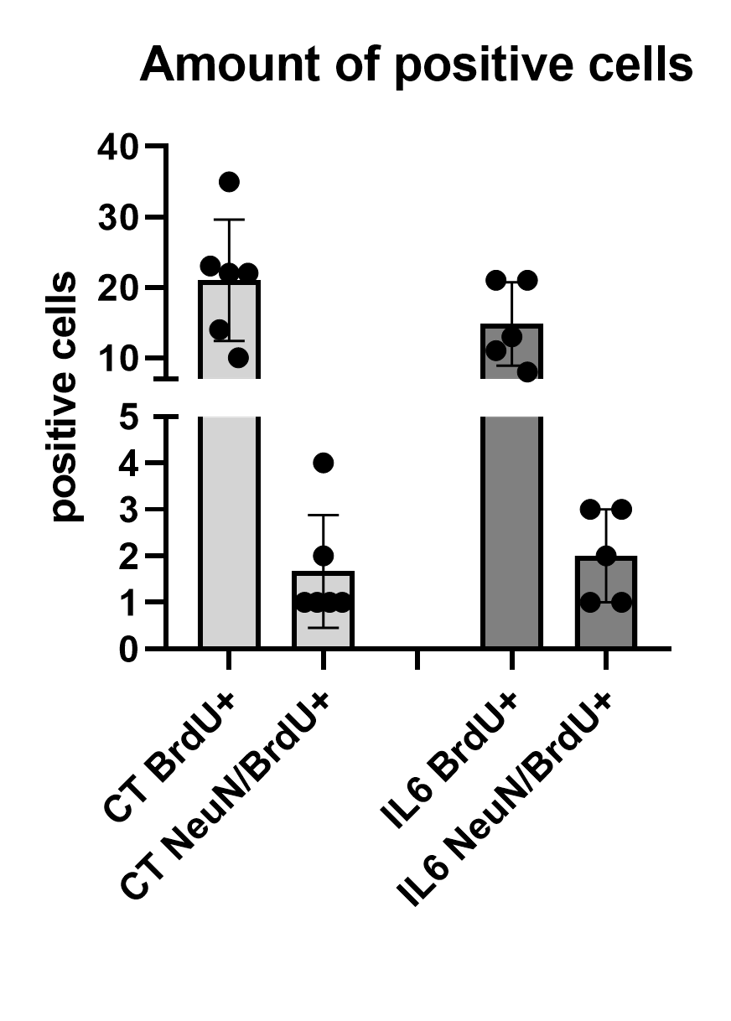
**

**Supplementary Table 1. Detailed information of primers used in the study.** All primers were purchased from Thermo Fischer Scientific.

| **Gene Name** | **Assay** | **Ref Seq** | **Exon Boundary** | **Assay Location** | **Amplicon Length** |
| --- | --- | --- | --- | --- | --- |
| Tnf | Mm00443258_m1 | [NM_001278601.1](http://www.ncbi.nlm.nih.gov/nuccore/NM_001278601.1) | 1-2 | 352 | 81 |
| Il1β | Mm00434228_m1 | NM_008361.3 | 3-4 | 160 | 90 |
| Il6 | Mm00446190_m1 | NM_031168.1 | 2-3 | 237 | 78 |
| Sox6 | Mm00488393_m1 | NM_001025559.3 | 8-9 | 1041 | 94 |
| Sox2 | Mm03053810_s1 | NM_011443.3 | 1-1 | 2343 | 86 |
| Dcx | Mm00438400_m1 | NM_001110222.1 | 2-3 | 617 | 100 |
| Gfap | Mm01253033_m1 | NM_001131020.1 | 6-7 | 1205 | 75 |
| Nestin | Mm00450205_m1 | NM_016701.3 | 2-3 | 1023 | 72 |
| Shh | Mm00436528_m1 | NM_009170.3 | 2-3 | 901 | 62 |
| Notch1 | Mm00627185_m1 | NM_008714.3 | 1-2 | 328 | 83 |
| Wnt5a | Mm00437347_m1 | NM_001256224.1 | 4-5 | 695 | 70 |
| Ctnnbl1 | Mm00499427_m1 | [NM_025680.4](http://www.ncbi.nlm.nih.gov/nuccore/NM_025680.4) | 7-8 | 879 | 64 |
| Bdnf | Mm01334043_m1 | NM_001285419.1 | 1-2 | 190 | 105 |
| Rax | Mm01258704_m1 | NM_013833.2 | 2-3 | 815 | 65 |
| Ascl1 | Mm03058063_m1 | NM_008553.4 | 1-2 | 1312 | 67 |
| Pcna | Mm05873628_g1 | NM_011045.2 | 1-1 | 227 | 72 |
| Agrp | Mm00475829_g1 | NM_001271806.1 | 3-4 | 447 | 86 |
| Pomc | Mm00435874_m1 | NM_001278581.1 | 3-4 | 461 | 60 |
| Npy | Mm00445771_m1 | NM_023456.2 | 2-3 | 259 | 65 |
| Cartpt | Mm04210469_m1 | NM_001081493.2 | 2-3 | 295 | 102 |
| Gapdh | Mm99999915_g1 | NM_001289726.1 | 2-3 | 117 | 107 |

**Supplementary Table 2. Raw data for statistical analysis.**
